# The effect of tumor necrosis factor-alpha on rheumatoid arthritis-related pain: overview of evidence and mechanistic pathways

**DOI:** 10.3325/cmj.2026.67.112

**Published:** 2026-04

**Authors:** Andrej Belančić, Seher Sener, Marija Rogoznica Pavlović, Mirjana Stanić Benić, Yusuf Ziya Sener, Almir Fajkić, Marijana Vučković, Josipa Radić, Mislav Radić

**Affiliations:** 1 Department of Basic and Clinical Pharmacology with Toxicology, Faculty of Medicine, University of Rijeka, Rijeka, Croatia; 2 Department of Pediatric Rheumatology, Sophia Children’s Hospital, Erasmus University Medical Center, Rotterdam, the Netherlands; 3 Hospital for Medical Rehabilitation of Heart and Lung Diseases and Rheumatism Thalassotherapia-Opatija, Opatija, Croatia; 4 Department of Internal Medicine, Dr. Josip Benčević General Hospital, Slavonski Brod, Slavonski Brod, Croatia; 5 Department of Cardiology, Thoraxcenter, Erasmus University Medical Center, CB Rotterdam, Rotterdam, the Netherlands; 6 Department of Pathophysiology, Faculty of Medicine, University of Sarajevo, Sarajevo, Bosnia and Herzegovina; 7 Department of Internal Medicine, Division of Nephrology and Dialysis, University Hospital of Split, Split, Croatia; 8 Internal Medicine Department, School of Medicine, University of Split, Split, Croatia; 9 Department of Internal Medicine, Division of Rheumatology, Allergology and Clinical Immunology, Center of Excellence for Systemic Sclerosis in Croatia, University Hospital of Split, Split, Croatia; The first two authors contributed equally and share the first authorship.

## Abstract

Rheumatoid arthritis (RA) is a chronic inflammatory disease characterized by debilitating pain and progressive joint destruction. Tumor necrosis factor-alpha (TNF-α), a key pro-inflammatory cytokine, has been implicated in modulating both peripheral and central pain pathways. TNF-α inhibitors, initially developed to halt structural damage in RA, have demonstrated substantial efficacy in pain relief, independent of their anti-inflammatory properties. This narrative review explores the mechanistic pathways through which TNF-α contributes to pain sensitization and highlights the role of TNF-α inhibitors in disrupting these pathways. Evidence suggests that these biologics not only reduce synovial inflammation but also modulate neuroinflammatory circuits, altering pain perception at the spinal and supraspinal levels. Despite their clinical success, variability in patient response and concerns regarding long-term safety necessitate further research into personalized therapeutic strategies. Identifying biomarkers predictive of pain relief could enhance treatment precision. Ultimately, the integration of TNF-α inhibitors into multidisciplinary pain management approaches holds promise for improving clinical outcomes and quality of life in RA.

Rheumatoid arthritis (RA) is the most common inflammatory joint disease worldwide. It is a complex, multifaceted, and persistent autoimmune disease. RA is characterized by chronic joint inflammation, resulting in symmetrical polyarthritis, synovial membrane hypertrophy, progressive joint deterioration, deformities, and destruction of bone and cartilage (1).

Although traditionally associated with inflammation, chronic pain in RA can persist beyond the clinical signs of disease and involves complex processes of peripheral and central sensitization. Chronic pain is common in patients with RA and can cause various physical and psychological impairments (2). Despite the availability of disease-modifying therapies, many patients continue to experience poorly managed pain (3,4). There is a need for a better understanding of the underlying mechanisms and optimal treatment strategies in RA.

Tumor necrosis factor α (TNF-α) is involved in pain modulation by promoting peripheral and central sensitization and increasing the excitability of nociceptors and neuroinflammation, as is the case in chronic inflammatory diseases such as RA (5). It also causes local inflammation and pannus formation. These processes lead to further cartilage erosion and bone destruction because they affect various cells in the synovial membrane, including synoviocytes, macrophages, chondrocytes, and osteoclasts (which can produce metalloproteinases, collagenase, and stromelysin) (6).

TNF-α inhibitors have transformed the treatment of RA as they provide a new level of disease control. They suppress synovial inflammation by neutralizing the ability of TNF-α to activate immune cells, stimulate osteoclasts, and promote leukocyte infiltration into joints, thereby affecting cartilage erosion and radiographic progression (7). They also reduce peripheral sensitization by neutralizing the role of TNF-α in synovial inflammation and osteoclast activation while reducing central sensitization by modulation of pain processing in the spinal cord and higher brain centers (8,9).

Administration of TNF-α inhibitors rapidly abolishes central nervous system (CNS) activity associated with nociceptive stimuli in patients with arthritis (10). This dual effect on inflammation and central pain processing makes these inhibitors an important potential therapeutic option for the treatment of inflammatory diseases with chronic pain. Additionally, TNF-α inhibition showed a reduction in subjective pain intensity ratings within 24 hours, which demonstrated its immediate impact on the CNS's pain responses, even before inflammation reduction is noticeable. Therefore, TNF-α inhibitors show promise for both acute and long-term pain management in RA (10).

This review aims to critically examine the analgesic efficacy of TNF-α inhibitors by looking at different mechanistic pathways. We also evaluated direct head-to-head comparisons and indirect evidence on this topic to consolidate the current knowledge on TNF-α inhibitors and their applicability in the management of pain associated with RA.

## Literature search and framing approach

This narrative review with scoping elements aimed to integrate mechanistic, preclinical, and clinical evidence on TNF-α-mediated pain pathways and the analgesic effects of TNF-α inhibitors in RA. PubMed and Scopus were searched for articles published from 2000 to 2025. Reference lists from relevant articles were manually screened. The search included earlier seminal studies where necessary to contextualize TNF-α biology and pain mechanisms. Search terms included “tumor necrosis factor-alpha,” “TNF-α inhibitors,” “rheumatoid arthritis,” “pain,” and related terms addressing neuroimmune mechanisms, nociception, cytokines, and patient-reported outcomes (PROs). Studies were considered eligible if they reported mechanistic or preclinical findings, clinical outcomes of TNF-α inhibitors (including pain and PROs), comparison with other RA therapies, or conceptual or translational insights into TNF-α and pain in RA. Non-English publications were excluded. Evidence was synthesized narratively and organized thematically to integrate biological mechanisms with clinical observations.

## Pathophysiology of pain in rheumatoid arthritis

RA is associated with a complex and diverse pain experience, which, depending on the underlying mechanisms, can be categorized as inflammatory, nociplastic (non-inflammatory), neuropathic, and nonarticular pain. Inflammatory pain arises from joint inflammation, typically accompanied by swelling, warmth, and morning stiffness, and generally responds well to anti-inflammatory treatments. This type of pain is primarily driven by pro-inflammatory cytokines such as TNF-α and interleukin-6 (IL-6), which activate immune cells and promote the release of other inflammatory mediators (11,12). In contrast, nociplastic pain is not directly linked to inflammation or structural joint damage but arises from altered central pain processing mechanisms. This type of pain often presents as widespread, even when inflammation is well-controlled, which suggests mechanisms of central sensitization, including enhanced excitability of nociceptive neurons in the CNS (13). Neuropathic pain, although less common in RA, results from nerve damage or compression and is characterized by burning, tingling, or electric shock-like sensations, requiring a tailored approach to pain management due to its distinct pathophysiology (14). Nonarticular pain is not confined to the joints but may affect broader areas such as the axial skeleton, muscles, or upper body regions, which influences remission rates in early RA. This type of pain may be persistent and may coexist with other chronic pain syndromes, further complicating the clinical picture (15). Recognizing these different types of pain is essential for effective management, as they require distinct therapeutic strategies. For example, inflammatory pain responds well to disease-modifying antirheumatic drugs (DMARDs) and biologics targeting the underlying inflammatory pathways. In contrast, nociplastic pain may benefit from nonpharmacological interventions, including cognitive-behavioral therapy, physical rehabilitation, and therapies targeting the CNS, such as serotonin-norepinephrine reuptake inhibitors or gabapentinoids (16).

**Figure 1:**
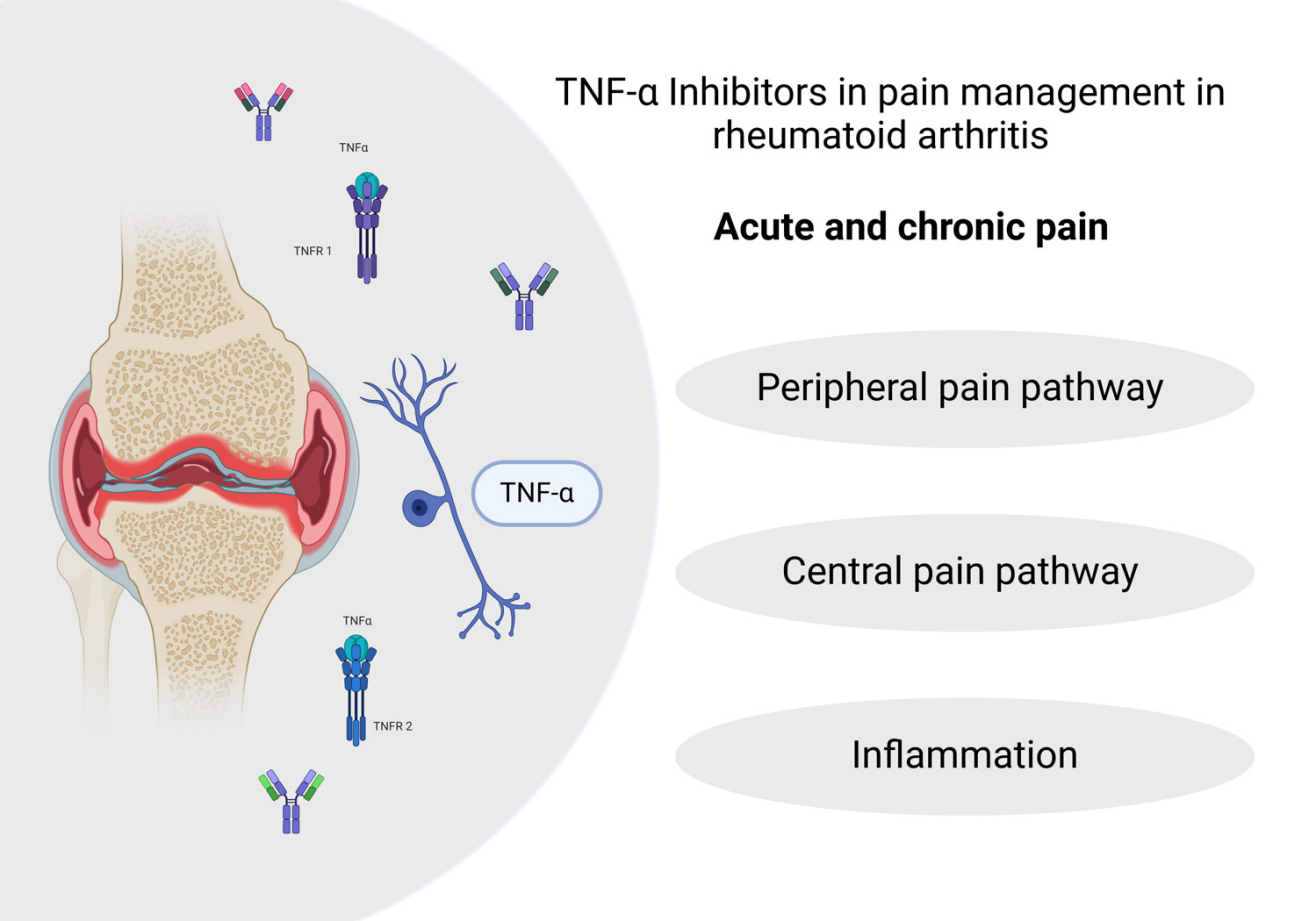
The role of tumor necrosis factor-alpha (TNF-α) inhibitors in rheumatoid arthritis-related pain. Abbreviations: TNFR – tumor necrosis factor receptor; CNS – central nervous system; IL-6 – interleukin 6; RA – rheumatoid arthritis.

Within this heterogeneous pain landscape, TNF-α is one of the key molecular mediators linking inflammatory activity with both peripheral nociceptor sensitization and central pain amplification. TNF-α plays a pivotal role in regulating both peripheral and central pain pathways in RA, acting as a pro-inflammatory cytokine that enhances synovial inflammation, peripheral nociceptor sensitization, and central sensitization (Figure 1). In the peripheral pain pathway, TNF-α is produced by synovial cells and infiltrating immune cells within inflamed joints, where it influences fibroblast-like synoviocytes and nociceptive neurons. This increases expression of pro-inflammatory genes and cytokines, promoting the depolarization and excitability of nociceptive neurons. This process includes enhanced transient receptor potential vanilloid 1 (TRPV1) function in sensory neurons and increased expression of receptors such as TRPV1 in dorsal root ganglion (DRG) neurons, contributing to thermal hyperalgesia (17,18). In the central pain pathway, TNF-α contributes to spinal cord hyperexcitability, a hallmark of central sensitization, by enhancing synaptic transmission and plasticity through its receptors TNFR1 and TNFR2. This modulation affects spinal neurons' excitatory synaptic currents and N-methyl-D-aspartate receptor activity, amplifying central sensitization and inflammatory pain. TNF-α application in the spinal cord increases the responsiveness of spinal neurons to nociceptive stimuli, while inhibition of TNF-α can reduce this hyperexcitability and relieve chronic pain (8,9).

Non-inflammatory pain in RA, commonly known as nociplastic pain, is primarily associated with alterations in central pain processing mechanisms. These changes persist even when inflammation is effectively managed, indicating a chronic pain state independent of peripheral joint damage. Nociplastic pain results from functional changes within the CNS, including increased neuronal excitability and disrupted pain modulation pathways, rather than being linked to peripheral inflammation or structural joint damage (16). An important feature of this type of pain is central sensitization, which enhances the sensitivity of nociceptive neurons to normal stimuli, leading to chronic pain despite the absence of active inflammation. This phenomenon is characterized by hyperalgesia and allodynia, where non-painful stimuli are perceived as painful, adding to the complexity of pain in RA (19,20). RA often coexists with fibromyalgia syndrome, which contributes to widespread pain through heightened central pain processing. It complicates clinical assessments by artificially increasing disease activity scores, which may not accurately reflect inflammatory activity (21). Neurogenic inflammation, driven by neuroendocrine mechanisms, can also sustain hyperalgesia by promoting the release of TNFα and IL-6, even in the absence of active synovitis (21,22). Additionally, impairments in descending pain modulation pathways reduce the body’s natural ability to regulate pain, as evidenced by weaker conditioned pain modulation in RA patients compared with healthy individuals. This impaired pain inhibition mechanism exacerbates pain perception and contributes to the persistence of chronic pain states (23). Psychosocial factors, including depression, anxiety, and sleep disturbances, further intensify pain perception and contribute to its chronic nature in RA (21).

Inhibition of TNF-α significantly affects synovial inflammation, peripheral nociceptors, and central sensitization in RA due to its involvement in both inflammatory and non-inflammatory pain mechanisms. TNF-α inhibitors such as etanercept and infliximab effectively reduce the infiltration of inflammatory cells and the production of other pro-inflammatory cytokines within the joint, decreasing synovial inflammation and inflammatory pain (24,25). TNF-α enhances the excitability of nociceptive neurons, promoting peripheral sensitization by upregulating receptors such as TRPV1 in DRG neurons, which contributes to thermal hyperalgesia (17,18). Additionally, TNF-α maintains spinal cord hyperexcitability, a feature of chronic pain in RA. TNF-α inhibitors reduce this hyperexcitability by diminishing the central effects of TNF-α, thereby lowering widespread pain sensitization beyond the affected joint (8). They also improve descending pain modulation, which is typically impaired in RA, thus positively affecting central pain processing mechanisms (26). By targeting TNF-α, these inhibitors reduce peripheral inflammation and modulate central pain pathways, offering comprehensive pain relief. This underscores the importance of understanding the multifaceted roles of TNF-α in RA pain mechanisms, which can inform more effective and personalized pain management strategies. Given the crucial role of TNF-α in pain pathways, TNF-α inhibitors have been explored as potential therapies for alleviating RA pain.

## TNF-α inhibitors and pain: mechanism beyond inflammation

### TNF-α inhibitors: differential effect on pain beyond joint swelling reduction

Pain resulting from local inflammation, chronic tissue damage, and nerve stimulation is an important target in the management of RA. The therapeutic effect of TNF-α blockade in RA is therefore attributed to both acute and chronic pain modulation. While chronic pain in RA is well-documented, the rapid effect of TNF-α blockade on pain is less pronounced. The rapid pain evolution as a result of TNF-α inhibition is related to central pain perception and sensation. Hess et al (10) demonstrated that TNF-α inhibition can reset CNS nociceptive activity within the first 24 hours of treatment in humans. Neuronal activity was suppressed in the thalamus, somatosensoric cortex, cingulate, and insular cortex of the limbic system contralateral to the affected joints within 24 hours of TNF-α inhibitor administration (10). These CNS regions are involved in pain perception and response, the subjective sense of the inner body, and the processing of emotions and memory. This effect of TNF-α blockade is rapid and reversible. The described functional CNS changes precede the improvement of the inflammatory markers (C-reactive protein, erythrocyte sedimentation rate) (10).

The role of TNF-α in pain beyond local joint inflammation has also been explored in neuropathic pain models and in other neuroinflammatory conditions. These data are not specific to RA but are relevant because they help illustrate the broader capacity of TNF-α to influence neuronal excitability, glial activation, and pain sensitization. In the context of RA, this is of particular interest given that some patients develop neuropathic features, including rheumatoid neuropathy, and that pain may persist even when overt synovial inflammation is reduced (27,28). Experimental studies support the role of TNF-α in pain hypersensitivity and neuroimmune signaling (29,30). However, clinical evidence from non-RA neuropathic conditions, including disc herniation-induced sciatica, is mixed, and such findings should not be directly extrapolated to RA pain mechanisms or treatment response (31-37). Therefore, these models are best viewed as mechanistic support for a broader neuroinflammatory role of TNF-α rather than as direct evidence for analgesic efficacy in RA.

### Mechanism affecting pain modulation, neuronal signaling, and central mechanism

The main factors contributing to clinical and laboratory manifestations of RA are inflammation and decreased apoptosis. TNF-α is involved in cell signaling and may modulate both inflammation and apoptosis. The inhibition of this key cytokine correlates with a decrease in inflammation and enhancement of the apoptosis of pro-inflammatory cells (38-40). Moreover, one of the main effects of TNF-α is maintaining a balance between cell survival and death, inducing an inflammatory response and apoptosis and necroptosis (38,41). Nerve injury, as a primary nociceptive stimulus, is closely related to endothelial damage and increased neuronal activity that favors local microglia transformation to macrophage-like cells capable of secreting TNF-α, tumor necrosis factor receptors (TNFRs), IL-1, and IL-6, which leads to heightened inflammation (42). Secreted glia-derived proinflammatory cytokines are involved in the activation of the p38-mitogen activated protein kinase system (p-38 MAPK) (42,43). Except for peripheral microglia mediation, primary afferent spinal neurons and spinal astrocytes are involved in signal conduction through the p-38 MAPK pathway, contributing to central enrollment of TNF-α. Spinal astrocytes, which are able to produce TNF-α, can be stimulated by produced TNF-α (44). Additionally, TNF-α is found in dorsal root ganglia sensory neurons and distant adjacent nerves, which supports its role as a key inflammatory mediator and a pain-modulatory factor (44). At the microenvironment level, TNF-α exerts its effect through stimulation of voltage-gated sodium channels and K^+^ membrane conduction in a non-voltage-gated fashion in sensory neurons (38,45). Simultaneous and exogenous inhibition, positive and negative feedback loops, mutual upregulation, and involvement of many other signal transducers make a neuroinflammatory signaling network very complex (39). Clinically, these mechanisms help explain why pain in RA may persist beyond the degree of visible joint inflammation and why TNF-α inhibition may provide benefit not only by reducing synovitis but also by modulating neuroimmune processes involved in peripheral and central sensitization.

### Preclinical and clinical biomarker studies on TNF-α and pain modulation

Preclinical studies, along with a limited number of small human studies, suggest that TNF-α may be involved in pain-related biological responses and could have potential as a biomarker candidate in selected clinical settings (46,47). However, current evidence does not support its use as an established clinical marker of pain response in humans. Human data remain heterogeneous with respect to disease context, sampling matrix, the timing of assessment, and correlation with pain intensity or clinical outcomes (48-56).

The potential clinical interest in TNF-α measurement lies in its possible role in improving pain phenotyping and, in selected contexts, supporting treatment stratification (57). Reported advantages include the availability of low- or minimally invasive sampling methods, including saliva, urine, and blood (48,49,58). Nevertheless, the current literature remains preliminary, and broader validation studies are needed before TNF-α can be considered a reliable clinical biomarker in routine practice.

Table 1 summarizes evidence from non-RA clinical settings, illustrating a broader biomarker potential of TNF-α rather than providing RA-specific validation (48-56). In a study by Grosman-Rimon et al (54), patients with acute myofascial pain syndrome had a significantly higher TNF-α level than healthy controls. Also, vascular endothelial growth factor (VEGF) levels were significantly correlated with TNF-α (r = 0.88, *P* < 0.001). This finding may support further investigation of TNF-α as a biomarker candidate in myofascial pain syndrome, but its diagnostic or therapeutic relevance remains to be established (54). A small cross-sectional study in patients with low back pain reported higher TNF-α and IL-6 levels than in controls, although the strength and generalizability of this observation are limited by the modest sample size (55). Patients who underwent corneal ablation surgery had a significantly higher concentration of salivary TNF-α levels post-surgery compared with baseline (52). No correlation between TNF-α and pain intensity was found (52). In elderly people with dementia, a statistically significant correlation between salivary TNF-α levels and pain response was found (51). In a study by Cantón-Habas et al, patients with chronic primary low back pain (LBP) had higher concentrations of urinary TNF-α compared with matched controls without chronic low back pain. Persistent pain was associated with higher levels of TNF-α than episodic pain. Moreover, baseline TNF-α concentrations and their changes after 4 weeks predicted alterations in pain intensity and disability following a spinal manipulative procedure (49). Klyne et al reported that higher and persistent TNF-α levels were associated with poorer 12-month recovery after acute LBP (50,53). The levels of salivary TNF-α were investigated in adults with cerebral palsy and healthy volunteers after an exogenous pain stimulus, an intramuscular injection. Although salivary TNF-α increased after injection in both groups, the groups did not differ in the correlation of salivary TNF-α and pain response (48).

**Table 1:** Studies investigating tumor necrosis factor-alpha as a biomarker in pain-related diseases

Author year/ reference ID	Study design	Participant/setting	Outcome	Results
Bazzichi et al, 2007 (56)	Descriptive study	80 patients with fibromyalgia (FM)	Plasma levels of interleukin (IL)-1, IL-6, IL-8, IL-10, tumor necrosis factor (TNF) -α, interleukin-6 (IL-6), rheumatoid factor, anti-extractable nuclear antigen antibodies, and anti-nuclear factor	The levels of IL-10, IL-8, and TNF-α were higher in FM patients than in controls.
Kraychete et al, 2010 (55)	Cross-sectional study	23 patients with low back pain (LBP) and 10 healthy controls	IL-1 β, TNF-α, IL-6, and soluble TNF-R	The levels of TNF-α and IL-6 were higher in patients with LBP.
Grosman-Rimon et al, 2016 (54)	Case-control study	37 patients with myofascial pain syndrome and 21 healthy controls	Blood levels of TNF-α, IL-6 and IL-12; IL-8, eotaxin, granulocyte-macrophage colony-stimulating factor, monocyte chemoattractant protein-1 (MCP-1), macrophage-derived chemokine (MDC), macrophage inflammatory protein(MIP)-1a, MIP-1b; IL-10 and IL-1 receptor antagonist (IL-1ra); and growth factors fibroblast growth factor-2 (FGF-2), vascular endothelial growth factor (VEGF), and platelet-derived growth factor (PDGF)	The levels of IL-6, TNF, and IL-12, MCP-1, MDC, eotaxin, GM-CSF, IL-8, and MIP-1b, IL-1ra and IL-10, FGF-2, PDGF, and VEGF were significantly higher in patients than in healthy controls.
Klyne et al, 2018 (53)	Prospective longitudinal study	109 patients with two weeks of onset of acute LBP and 55 pain-free controls	C-reactive protein (CRP), tumor necrosis factor (TNF), IL-6, and IL-1β, and questionnaires related to pain, disability, sleep, and psychological status	CRP was higher in acute LBP than in controls at baseline. In LBP patients, baseline CRP was higher in the recovered than in the non-recovered groups. Conversely, TNF was higher at both time points in the non-recovered than in the recovered groups. Two sub-groups were identified that associated with more “inflammatory/ poor sleep” or less “high TNF/ depression” recovery.
Sobas et al, 2020 (52)	Multicenter, prospective, and descriptive cohort study.	32 patients (19 men and 13 women) underwent corneal advanced surface ablation surgery	Pain intensity before and after surgery assessed by visual analog scale (VAS) and the numeric pain rating scale. Cortisol, salivary α amylase, salivary IgA, testosterone, and salivary TNFαRII	The concentration of salivary IgA and salivary TNFαRII post-surgery was significantly higher compared with baseline (*P* value: 0.053). Relations between VAS scale score and putative biomarker variations were not statistically significant.
Cantón-Habas et al, 2021 (51)	Cross-sectional study	75 elderly patients diagnosed with dementia and a global deterioration scale score of 5 to 7	The Pain Assessment in Advanced Dementia (PAINAD) scale and the collection of salivary TNF-RII and salivary IgA	A correlation between the PAINAD scale (numeric, binary, and recoded) and sTNF-RII and sIgA (*P* < 0.001).
Klyne et al, 2022 (50)	Longitudinal cohort study	83 patients with 2 weeks of an acute episode of LBP – grouped into 4 clusters: “inflammatory and poor sleep” (Cluster 1), “high TNF and depression” (Cluster 2), “high pain and high pain-related fear” (Cluster 3), and “low pain and low pain-related fear” (Cluster 4)	Self-reported LBP (0-10 numerical rating scale), LBP-related disability (Roland-Morris Disability Questionnaire), depression (Center for Epidemiological Studies Depression Scale), pain catastrophizing (Pain Catastrophizing Scale), fear avoidance (Fear Avoidance Beliefs Questionnaire), pain self-efficacy (Pain Self-Efficacy Questionnaire), and sleep (Pittsburgh Sleep Quality Index); systemic inflammatory biomarkers (CRP, IL-6, IL-1β, TNF)	Over 12 mos, Cluster 1 reported the greatest recovery at every 3-monthly interval, whereas Cluster 2 reported the least recovery. Cluster 1 had elevated CRP (and IL-6) at baseline that continued to decrease from 3 to 9 mos. TNF was elevated early and persistently in Cluster 2.
Gevers-Montoro et al, 2023 (49)	Case-control study	24 patients with chronic primary low back pain (CPLBP) and 24 sex- and age-matched asymptomatic controls	Urinary TNF-a measured at baseline and after 4 weeks, during which CPLBP patients underwent spinal manipulative therapy (SMT)	The concentrations of TNF- α were higher in baseline urine samples of CPLBP patients compared with controls. Patients with persistent pain showed higher levels of TNF-α than those with episodic CPLBP. Baseline TNF-α concentrations and their changes after 4 weeks predicted alterations in pain intensity and disability following SMT in patients with CPLBP.
Sabater-Garriz et al, 2024 (48)	Case-control study	30 patients with cerebral palsy and 30 healthy volunteers	Salivary levels of sTNF-α, sIgA, cortisol, ferric reducing antioxidant power, adenosine deaminase, and alpha amylase, and 3 observational pain scales (Wong-Baker, Non-Communicating Adults Pain Checklist, and Facial Action Coding System) after an exogenous pain stimulus, an intramuscular injection	Pain in patients with CP was higher than in healthy controls after the intramuscular injection. sTNF-α showed a significant post-stimulus increase in both groups.

TNF-α exerts its effect as a proinflammatory cytokine and pain modulator at the local and central level in a very intricate manner. It is only one important piece of a complex puzzle in the network signaling pathway related to pain modulation. Overall, existing reports suggest potential biomarker relevance of TNF-α in pain-related conditions, but the evidence remains preliminary and insufficient for firm predictive clinical use. Most currently available biomarker studies have been conducted in non-RA settings, which limits the direct translation of these observations to RA. Although existing reports are encouraging, current data are insufficient to support firm predictive or diagnostic clinical use in RA, and more rigorous, standardized, and disease-specific studies are needed.

### Clinical evidence: TNF-α inhibitors for pain relief

TNF-α is a key mediator in the pathogenesis of synovial inflammation, pain, and systemic manifestations of RA (59). TNF-α inhibitors neutralize TNF-α and attenuate its pro-inflammatory effects, and they represent an established class of biologic therapies for RA (60,61). Clinical studies support their efficacy in reducing pain in many patients with RA (62). In addition to reducing inflammation, TNF-α inhibitors may influence pain processing through mechanisms not fully explained by structural outcomes alone, although the extent and clinical relevance of these effects remain to be more clearly defined (63).

### Randomized controlled trials evaluating pain outcomes in rheumatoid arthritis

Randomized trials have shown that TNF-α inhibitors improve pain outcomes in RA, particularly when assessed alongside broader measures of disease activity and physical function (64-67). In the ATTRACT trial, infliximab combined with methotrexate was associated with greater pain improvement than methotrexate alone (67). In the TEMPO trial, combination therapy with etanercept and methotrexate resulted in superior PROs, including pain, compared with either agent used as monotherapy (66). In methotrexate-naïve patients, golimumab also demonstrated early and sustained clinical benefit, including improvement in pain-related outcomes (65). The EXXELERATE trial further showed that adalimumab and certolizumab pegol produced broadly comparable outcomes, without clear evidence of superiority of either TNF-α inhibitor (64).

### Meta-analyses and long-term observational studies on pain relief

Meta-analyses and long-term observational studies offer valuable insights into sustained effects and real-world effectiveness of TNF-α inhibitors in RA pain management. A meta-analysis of 31 RCTs found significant reductions in pain associated with TNF-α inhibitors, further solidifying their role as effective agents in pain management for RA (68). Another systematic review and meta-analysis found that TNF-α inhibitors effectively reduced RA activity, alleviated pain, and improved physical function, especially in patients with moderate to severe disease unresponsive to conventional therapies (69). These findings underscore the effectiveness and generally favorable safety profile of TNF-α inhibitors, highlighting their sustained long-term benefits in the management of RA.

Regarding observational studies, the BIOBADASER study demonstrated a significant reduction in pain among patients treated with TNF-α inhibitors, establishing their effectiveness in real-world settings (70). Another observational study assessed pain reduction in RA patients treated with TNF-α inhibitors over six months. The pain reduction was measured using standardized pain scales, and patients with RA experienced notable improvement in pain levels compared with baseline, with TNF-α inhibitors contributing to enhanced disease control and overall symptom relief (71). Kievit et al (72) demonstrated that TNF-α inhibitors provided sustained pain relief throughout treatment, with significant improvements in RA activity and overall symptom management. Additionally, the study observed long-term benefits in pain reduction and physical function.

### Clinical considerations that may influence pain outcomes across TNF-α inhibitors

Although TNF-α inhibitors share a similar mechanism of action, they differ in their effectiveness, pharmacokinetics, and tolerability profiles. These differences help guide clinicians in selecting the most appropriate agent based on individual patient needs and treatment goals.

Infliximab is notable for its rapid onset of action, which makes it especially effective for patients with severe RA. Its intravenous delivery allows rapid absorption, potentially leading to faster clinical improvement, which can be crucial in managing acute flare-ups or severe disease states (73). Etanercept, in contrast, offers consistent and reliable pain relief and is generally associated with fewer infusion-related reactions compared with infliximab. Etanercept’s subcutaneous administration may be more convenient as it reduces the need for frequent hospital visits for intravenous infusions (74).

Adalimumab provides sustained pain relief and is associated with high levels of patient adherence, attributable to its subcutaneous administration and ease of use at home. Its favorable safety profile also supports long-term adherence, making it a preferred choice for many patients (75).

Golimumab, administered once a month, provides moderate pain relief. Its less frequent dosing schedule is attractive for patients, particularly those with busy lifestyles, as it minimizes the need for frequent injections and can improve adherence while reducing the burden of ongoing treatment (76).

Certolizumab pegol is effective in patients with RA who have not responded to other treatments (77). It targets a specific protein involved in the inflammation process. The unique structure of certolizumab pegol, which includes a polyethylene glycol moiety, enhances its pharmacokinetics and leads to prolonged effectiveness. In clinical trials, certolizumab pegol has demonstrated significant pain reduction and improved clinical outcomes, particularly in difficult-to-treat patients (78). This underscores its importance in the management of RA when other therapies have failed.

### The role of biosimilars in pain relief

Biosimilars, biologic drugs that are highly similar to reference TNF-α inhibitors, have emerged as a cost-effective alternative to original biologics without compromising therapeutic efficacy (79). These drugs mirror the properties of their reference counterparts, offering the same clinical benefits, including pain relief, at a lower cost (79).

In the PLANETRA trial, an infliximab biosimilar effectively reduced inflammation and provided pain relief comparable to the reference drug in RA patients (80). The NOR-SWITCH trial further explored the efficacy of biosimilars in real-world settings by examining the effects of switching from the originator infliximab to its biosimilar. After the switch, no significant loss of pain relief efficacy was found, which indicates that patients who transition from a reference drug to its biosimilar maintain similar levels of pain control (81). These findings support the safety and effectiveness of biosimilars in clinical practice, particularly for patients who may benefit from the reduced costs associated with biosimilars.

### Patient-reported outcomes: real-world data on pain control

Real-world evidence and PROs offer invaluable insights into pain management that extend beyond the controlled environment of clinical trials (82). These data sources enable a more comprehensive understanding of treatment efficacy, not only through assessing objective clinical measures but also by considering patients' personal experiences, treatment satisfaction, and the impact of therapy on their quality of life (82). TNF-α inhibitors have gained recognition for their effectiveness in alleviating pain; however, patient adherence and persistence rates vary. This variability can be attributed to factors such as individual responses to the drug, the presence of side effects, and the overall impact on quality of life during treatment.

A real-world study on TNF-α inhibitors, including etanercept, adalimumab, and infliximab, demonstrated significant pain reduction, improved disease control, and enhanced quality of life in RA patients (83). Long-term use of these biologic therapies was associated with sustained pain relief, and treatment persistence was higher among patients who experienced substantial pain reduction. Another study found that TNF-α inhibitors effectively reduced pain and improved physical function, as reflected by improved Health Assessment Questionnaire (HAQ) scores (84). Both studies highlight the clinical benefits of TNF-α inhibitors in pain relief and physical function improvement in real-world clinical practice.

## TNF-α inhibitors vs other rheumatoid arthritis treatments for pain

Although TNF-α inhibitors are essential in treating RA, other options also play important roles in RA management. These include nonsteroidal anti-inflammatory drugs (NSAIDs), corticosteroids, methotrexate, Janus kinase (JAK) inhibitors, and IL-6 inhibitors (85). NSAIDs and corticosteroids provide rapid symptom relief, while conventional DMARDs such as methotrexate remain a cornerstone of RA therapy (86). In recent years, targeted synthetic DMARDs such as JAK inhibitors, and alternative biologic therapies, including IL-6 inhibitors, have become effective options for patients who inadequately respond to TNF-α inhibitors (85). The choice of therapy should be guided by patient-specific factors, including disease severity, treatment response, safety profile, and comorbidities, to achieve optimal pain management while minimizing adverse effects. The effectiveness of pain relief may differ among drug classes.

### JAK inhibitors vs TNF-α inhibitors

JAK inhibitors, including tofacitinib and baricitinib, target intracellular signaling pathways that regulate immune responses (87). In contrast to TNF-α inhibitors, which act extracellularly, JAK inhibitors modulate inflammatory signaling more directly (87). Clinical trials indicate that JAK inhibitors provide rapid pain relief, sometimes within days, compared with a weeks-long delay seen with TNF-α inhibitors (88,89). For instance, the ORAL Strategy trial demonstrated that tofacitinib was noninferior to adalimumab in terms of pain reduction and disease control in patients with RA who had an inadequate response to methotrexate (89). Another study found that baricitinib significantly reduced pain and improved disease control in moderate-to-severe RA patients who had failed at least one TNF-α inhibitor, with a faster onset of relief than TNF-α inhibitors (88). Accordingly, baricitinib could serve as a valuable alternative for patients who do not respond adequately to TNF-α inhibitors.

### IL-6 inhibitors vs TNF-α inhibitors

IL-6 inhibitors, such as tocilizumab and sarilumab, target a key mediator of inflammation that plays a crucial role in the pathogenesis of RA (90). Unlike TNF-α inhibitors, which primarily target TNF-α, IL-6 inhibitors provide an alternative pathway for controlling the inflammatory cascade in RA patients, particularly in those with high systemic inflammation (91). Some studies suggest that IL-6 inhibitors may be superior to TNF-α inhibitors in controlling pain in patients with high systemic inflammation (92,93). A head-to-head RCT demonstrated that tocilizumab monotherapy surpassed adalimumab monotherapy in reducing RA-related pain and enhancing physical function (93). Similarly, the MONARCH trial found sarilumab to be more effective than adalimumab in alleviating RA-related pain (92).

### Nonsteroidal anti-inflammatory drugs, methotrexate, and corticosteroids

NSAIDs are used during the acute phase response to alleviate pain by mitigating inflammation. They achieve their pharmacological effects by inhibiting cyclooxygenase (COX), particularly COX-2, which increases during inflammatory responses. Nonetheless, the potential for injury must be acknowledged, as the suppression of prostaglandins may result in significant adverse effects. Certain negative effects may be mitigated by utilizing COX-2-selective NSAIDs (94,95). The efficacy of NSAIDs in RA has been established by placebo-controlled trials with patients not receiving glucocorticoid treatment (96). Consequently, they are often used as an adjunct therapy rather than as a primary treatment.

Glucocorticoids (GCs) exhibit superior potency and efficacy compared with NSAIDs, attributable to the intricate processes underlying their anti-inflammatory and immunosuppressive actions; nonetheless, the safety profile of NSAIDs is somewhat more favorable (97). GCs have two primary functions in the management of RA: they work as bridge therapy for DMARDs until their therapeutic benefits manifest, and they function as supplementary therapy for persistent active RA despite DMARD use (98). The SEMIRA trial investigated the tapering of GSc in patients on tocilizumab, demonstrating that maintaining IL-6 inhibition allowed for steroid dose reduction while sustaining pain relief (99).

DMARDs are used to induce remission by inhibiting autoimmune activity and delaying or preventing joint deterioration and pain (98). Treatment should commence promptly. Conventional synthetic DMARDs (csDMARDs) are generally employed as the initial treatment for newly diagnosed RA patients (100). The 2021 American College of Rheumatology (ACR) recommendation for RA treatment designates methotrexate as the primary therapeutic option, both as a monotherapy and in combination with other agents, due to its efficacy, safety profile, flexible administration, and affordability (101). The guideline strongly recommends methotrexate monotherapy over hydroxychloroquine, sulfasalazine, and biologics for DMARDs-naive RA patients with moderate-to-high inflammatory activity. Methotrexate monotherapy provided significant pain relief in early RA patients but was less effective than biologics in established disease (102). The early addition of biologics to methotrexate led to superior pain outcomes compared with step-up therapy (103). Additionally, the guideline conditionally recommends methotrexate over leflunomide (LEF) for RA patients who have not received DMARDs, and methotrexate monotherapy over dual or triple csDMARDs therapy, as well as over methotrexate combined with biologics. A meta-analysis revealed comparable efficacy and safety profiles of LEF and methotrexate (104).

### Combination therapy strategies

The efficacy of combination therapy, comprising TNF-α inhibitors and methotrexate or IL-6 inhibitors, in enhancing pain management has been investigated (85). Studies have shown that combining agents with different mechanisms of action can provide better pain control, especially in case of refractory symptoms. The BeST study demonstrated that early, intensive combination therapy enhanced pain and disease control when compared with step-up therapy (105). The SWEFOT trial compared step-up therapy using methotrexate with the addition of either TNF-α inhibitors or sulfasalazine/hydroxychloroquine. The results showed that patients receiving TNF-α inhibitors experienced faster pain relief and sustained improvement over time (103). Additional evidence from the AMPLE trial, which compared a combination of abatacept and methotrexate to TNF-α inhibitors with methotrexate, showed similar pain reduction but fewer injection-site reactions in the abatacept group (106).

## Head-to-head comparisons and indirect evidence

### Direct comparative trials

Peer-reviewed, published, direct head-to-head trials are scarce (107). Despite their known drawbacks and inconsistencies, indirect comparisons such as network meta-analysis have replaced head-to-head trials as the standard, even in treatment recommendations (107). Compared with placebo-controlled studies, direct head-to-head trials comparing effective therapies for RA can be more clinically relevant since they provide evidence-based therapy comparisons (108). The main obstacle to conducting direct trials is the huge sample size needed to show that the differences between treatments (109).

The EXXELERATE study was the first prospective trial to compare the effectiveness of certolizumab pegol and adalimumab, two TNF-α inhibitors, with a primary superiority endpoint at 12 weeks and 2 years in RA patients receiving background methotrexate (64). Certolizumab pegol did not demonstrate superiority over adalimumab, with identical ACR-20% response rates at week 12 (64,109). Additionally, the trial evaluated the effectiveness of single-blind switching to a second TNF-α inhibitor (without washout) in patients who had not initially responded to either adalimumab or certolizumab pegol. After 12 weeks, 58% of patients who switched from adalimumab to certolizumab pegol and 62% who switched from certolizumab pegol to adalimumab achieved DAS28-ESR≤3.2 or a decrease of 1.2 or more (64,109).

Other studies were designed to include biologics from classes other than TNF-α inhibitors. For example, ADACTA study compared the efficacy of tocilizumab vs adalimumab (107).

### Do differences in pharmacokinetics influence pain relief?

All five drugs that target TNF-α have different pharmacokinetics and routes of administration; each of them is unique in terms of clinical efficacy and tolerance (110). There is significant inter-individual variation in clinical response, which is impacted by pharmacokinetics (111,112). The current understanding of TNF-α inhibitors' pharmacokinetic variability has shown that a decrease in monoclonal antibody exposure is associated with both the presence of anti-drug antibodies and an increased antigenic burden, which leads to higher disease activity (110). Higher TNF-α inhibitor dosages would be required for RA patients based on the high disease activity (113). The dosages of TNF-α inhibitors administered intravenously can be adjusted to account for body size and disease activity. This is not the case for subcutaneous formulations, which are often produced with a predetermined dose regimen (eg, adalimumab 40-mg prefilled syringes) (110). Similar to Crohn's disease patients, for whom loading doses have improved treatment efficacy and lowered the chance of developing anti-drug antibodies, RA patients should benefit from a 160 mg loading dose (114).

Studies investigated the efficacy, safety, and pharmacokinetics of TNF-α inhibitors, but did not address the impact on pain (115,116). We can conclude that by acting on disease activity, TNF-α inhibitors indirectly affect pain.

### Network meta-analyses and indirect comparisons of pain outcomes

Meta-analyses of randomized controlled trials generally report efficacy using the standard primary outcomes of the ACR-20, 50, or 70 score (68). The ACR 20/50/70 score is a composite metric; it is difficult to determine which way significant patient-centered components will be affected. Physical function, which included a pain scale, was assessed using the Health Assessment Questionnaire Disability Index (HAQ-DI). From the perspective of the patient, PROs assess health, quality of life, and responsiveness to treatment (117). PROs that significantly affect RA patients' quality of life include pain, fatigue, work performance, and everyday activities (118).

Early therapy intervention with etanercept + methotrexate produced noticeably larger benefits in the majority of PROs (including physical functioning, weariness, pain, and general health state) than methotrexate alone (119). In the Trial of Etanercept and Methotrexate with Radiographic Patient Outcomes research, 44% of RA patients on etanercept + methotrexate achieved a low HAQ score (120).

Over the two years of the AMPLE study, patients treated with subcutaneous abatacept or adalimumab while receiving background methotrexate experienced similar, clinically significant improvements in four PROs: fatigue, pain, ability to complete work, and ability to perform daily activities (106). These improvements also had a similar beginning of response.

In the PREMIER study, patients with early RA treated with adalimumab plus methotrexate reported significantly better results from baseline to year 2 in HAQ DI (*P* < 0.0001), Short Form-36 health survey physical component summary score (*P* < 0.0001), patient global assessment score (*P* < 0.0001), and pain score (*P* < 0.0001) compared with patients treated with methotrexate monotherapy (121).

St Clair et al showed that methotrexate and infliximab together, when used to treat early RA, improved physical functioning (according to the HAQ score), reduced the radiographic development of joint damage, and improved signs and symptoms of disease activity more than methotrexate therapy alone during a one-year period (122). In RAPID 1&2 trials, certolizumab pegol with methotrexate significantly outperformed methotrexate plus placebo, both clinically and statistically, in terms of reducing pain and fatigue (123). In patients with active RA, golimumab injections administered every four weeks in addition to methotrexate dramatically decreased RA symptoms and enhanced physical function (124).

### Impact of dosing, administration route, and duration on pain response

When choosing a biologic drug, RA patients' self-efficacy in administering injections is considered significant (125). Patients may experience pain and severe skin reactions from injectable formulations; therefore, it is crucial to advise them about injection tolerance. Clinical trials have revealed a 15%-20% incidence of these symptoms, while data from a survey of 113 community-based rheumatologists' patients showed a substantially greater prevalence, almost 60%, with 22% of the population expressing moderate to severe pain (126). There was no difference in injection/infusion-site burning and stinging (ISBS) between etanercept and adalimumab, unless the distribution method (autoinjector, syringe, or pre-filled syringe) was considered (126,127). Navarro-Millan et al showed that adalimumab was less tolerated than etanercept in relation to ISBS (128). Although there is no definitive reason for the variations in ISBS observed with these biologics, the pH or other inactive substances may be contributing factors (128). The risk of ISBS was not significantly affected by the duration of biologic therapy (<6 months vs >6 months, or <12 months vs >12 months) (126).

ISBS is a localized peripheral nociceptive phenomenon (129), and repeated exposure to such stimuli may lead to sustained nociceptive input and potentially affect central pain processing, thereby indirectly influencing pain modulation mechanisms in RA.

## Unmet needs and future directions

### Why do some patients fail to achieve pain relief despite TNF-α inhibition?

In RA, the processes underlying chronic pain are still poorly understood (130). Pain may resist when RA is in remission and can occur at non-inflammatory locations (131,132). Patients with RA experience a variety of comorbidities besides their pain, such as fatigue and depression. Depression is associated with changes in several brain areas, including the hippocampus, which is also involved in the pain matrix (133).

In the absence of inflammation, persistent behavioral symptoms (such as depression) and pain indicate that alterations in the CNS brought on by inflammatory episodes persist over time, leading to abnormalities in psychology (134). Quantitative measurements of joint inflammation do not correlate with the level of pain experienced by patients (135,136).

TNF-α inhibitors, the cornerstone of modern RA treatment, tend to work less well for patients with chronic depression (137). Reduced pain tolerance linked to depression may lead to sensitization and interfere with endogenous pain inhibition, which might shape pain responses and clinical outcomes over time (138).

### Predictive biomarkers for response to TNF-α inhibitors

Based on a meta-analysis of several earlier studies, a systematic literature review published in 2017 found that treatment response to TNF-α inhibitors in RA patients was linked to genetic variations in *CHUK, PTPRC, TRAF1/C5, NFKBIB, FCGR2A*, and *IRAK3*. Additionally, genome-wide association studies (GWAS) found five associated genes (139). The 12 most promising single-nucleotide polymorphisms found in the three largest GWAS were not linked to TNF-α inhibitor response (140). When data were added to earlier findings and stratified by medication type, a variable mapping to *NUBPL* was linked to response to etanercept (140). Other research has considered genetic variations known to impact gene activity; for example, one study used machine learning techniques to reveal that TNF-α inhibitor response was associated with CD39 and CD40 (141). But altogether, genotypic scores only explained 1% of the variation in responses. Remarkably, a small study of 325 patients receiving adalimumab was linked to a connection with CD40LG, indicating that this could be a significant route in response to TNF inhibitor efficacy in RA (142). The authors identified associations with *TANK, VEGFA,* and *TNFAIP3* in addition to concentrating on 223 variations that map to inflammatory pathways (142). A molecular signature in peripheral blood mononuclear cells characterized by impaired function of *PTPN22*, a gene known to be associated with susceptibility to RA, has also been observed in active RA patients and their healthy first-degree relatives (143). Although studies have proved unsatisfactory due to inconsistent results, genetic markers of TNF-α inhibitor responsiveness are still investigated.

### Emerging TNF-α inhibitors-targeted pain therapy and other biologics

TNF-α inhibitors reduce inflammation-causing cell recruitment, provide rapid symptom relief, enhance function, lessen pain, and slow the progression of joint deterioration in RA (143). Early pain improvement after TNF-α inhibition has been reported in some patients, supporting the concept that these agents may influence pain processing through mechanisms not solely attributable to delayed anti-inflammatory effects (12). Over a 24-week period, TNF-α inhibitors and tocilizumab monotherapy both showed noticeably higher pain reductions (on a 0-100 scale) than placebo (144).

Clinical studies in patients unresponsive to methotrexate revealed that tocilizumab considerably reduced fatigue and pain as assessed by a visual analog scale (145,146). Anti-IL-6 or anti-IL-6 receptor medications reduced allodynia and hyperalgesia, and meta-analysis data validate that IL-6 can be targeted to reduce inflammatory pain (147).

In addition to reducing synovitis, anakinra and rituximab typically reduce joint pain in RA within two to four weeks of starting treatment (12). JAK inhibitors may also directly affect pain (148).

### Personalized medicine approaches in RA pain management

Pain remains a considerable problem in RA, despite major therapeutic advancements that have reduced synovitis and disease activity (149). Because pain in RA is a complex experience caused by multiple mechanisms, its management requires coordinated pharmacological, psychological, and physiotherapeutic approaches, along with a thorough evaluation of the condition’s causes and effects on each patient (12). The RA recommendations ought to place more emphasis on pain management (130).

The interplay of psychological variables, peripheral inflammation, and central sensitization emphasizes the necessity of a multifaceted approach to pain assessment and management (11). Characterization of comorbidities, RA pain causes, and concurrent medications helps choose the treatments with the best risk-benefit ratios for the patient (12). When examining individual responses to analgesia, one should consider placebo effects and regression to the mean, which are believed to account for almost half of analgesic efficacy in RCTs of analgesic therapies in RA (150). Individuals frequently place different relative values on risks and benefits. RA patients should base their use of analgesics on well-informed decisions (12). A thorough comprehension of these intricate pain dynamics helps direct focused treatment approaches and enhance the quality of life of inflammatory arthritis patients in general (11).

## Study limitations

As a narrative review, this study does not adhere to formal systematic review methodologies and does not include structured risk-of-bias assessment; therefore, potential selection bias and heterogeneity of included studies should be acknowledged. Nevertheless, this approach is appropriate for synthesizing diverse evidence and providing a comprehensive, hypothesis-driven overview, in line with narrative review reporting principles.

## Conclusion

TNF-α plays a pivotal role in the pathophysiology of pain and inflammation in RA, influencing both peripheral and central pain pathways. The advent of TNF-α inhibitors has revolutionized RA management, not only by mitigating joint destruction but also by modulating pain perception /alleviating pain, reducing neuroinflammation, and restoring immune homeostasis. Emerging evidence suggests that beyond their anti-inflammatory effects, TNF-α inhibitors exert direct analgesic actions, potentially altering pain-processing networks in the CNS. However, there remain challenges in terms of variability in patient response, risk of adverse effects, and the need for precision medicine approaches. A deeper understanding of TNF-α’s role in pain neurobiology could pave the way for novel therapeutic interventions, optimizing patient outcomes and enhancing quality of life for individuals with RA. Future research should focus on identifying specific biomarkers that predict individual responses to TNF-α inhibitors, which could enable more personalized treatment strategies and integrating these agents into comprehensive pain management for RA.
